# Go for Gold: Development of a Scalable Synthesis of [1‐(Ethoxycarbonyl)cyclopropyl] Triphenylphosphonium Tetrafluoroborate, a Key Reagent to Explore Covalent Monopolar Spindle 1 Inhibitors

**DOI:** 10.1002/open.202500106

**Published:** 2025-04-16

**Authors:** Leon Rebhan, Rebekka Fürst, Dieter Schollmeyer, Ricardo A. M. Serafim, Matthias Gehringer

**Affiliations:** ^1^ Department of Pharmaceutical/Medicinal Chemistry Institute of Pharmaceutical Sciences Eberhard Karls University Tübingen 72076 Tübingen Germany; ^2^ Department of Chemistry, Biochemistry and Pharmacy University of Bern 3012 Bern Switzerland; ^3^ Department for Medicinal Chemistry Institute for Biomedical Engineering Faculty of Medicine Eberhard Karls University Tübingen Auf der Morgenstelle 8 72076 Tübingen Germany; ^4^ Cluster of Excellence iFIT (EXC 2180) ‘Image‐Guided & Functionally Instructed Tumor Therapies’ University of Tübingen 72076 Tübingen Germany; ^5^ Department Chemie, Zentrale Analytik Johannes Gutenberg‐Universität Mainz 55128 Mainz Germany; ^6^ Department of Organic and Pharmaceutical Chemistry School of Engineering Institut Químic de Sarrià (IQS) Universitat Ramon Llull (URL) Vía Augusta 390 08017 Barcelona Spain

**Keywords:** covalent inhibitors, ethoxycarbonylations, phosphonium salts, protein kinase inhibitors, Wittig reactions

## Abstract

Covalent approaches have resurged in drug discovery and chemical biology during the last decade. So‐called targeted covalent inhibitors typically show a strong and persistent drug–target interaction as well as a high degree of selectivity. In our research group, **RMS‐07 (8)**, a First‐in‐Class covalent inhibitor of the protein kinase threonine tyrosine kinase (TTK)/monopolar spindle 1, which shows promising results in a variety of different solid cancer cell types and will be further optimized in terms of covalent binding kinetics, has recently been developed. However, synthetic accessibility is restricted by a high price and limited availability of [1‐(ethoxycarbonyl)cyclopropyl] triphenylphosphonium tetrafluoroborate (**10**), a key reagent required to assemble the tricyclic core scaffold in a Wittig‐type cyclization reaction. This reagent is also described as a valuable synthon for the synthesis of a range of ring systems with interesting applications in medicinal chemistry. However, reliable procedures for its large‐scale synthesis are scarce. Only one prior report describes the synthesis of reagent **10**, and it contains limited experimental details, making it challenging to reproduce and scale up. Herein, a concise and reproducible decigram‐scale synthetic protocol for accessing key reagent **10** is described.

## Graphical abstract

[1‐(Ethoxycarbonyl)cyclopropyl] triphenylphosphonium tetrafluoroborate is a limiting key reagent for synthesis of the tricyclic 5,6‐dihydropyrimido[5,4‐*e*]indolizine and related scaffolds, which are central to First‐in‐Class covalent inhibitors of the protein kinase monopolar spindle 1/threonine tyrosine kinase (TTK). Herein, a high‐yielding and reproducible decigram synthesis of this reagent enabling the exploration structure–activity relationships is described.

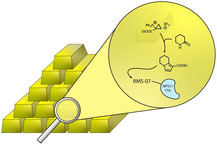

## 1 Introduction

**Figure 1:**
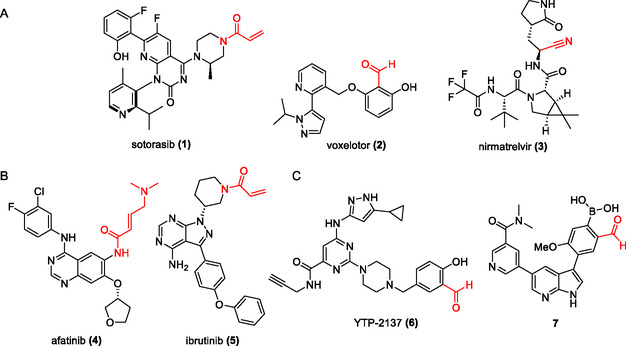
Examples of approved drugs (A and B) and high‐quality chemical probes (C) with a covalent mechanism of action. Warhead groups highlighted in red.

In the last decade, medicinal chemists have revived the interest in covalent strategies for drug discovery and chemical biology applications.^[^ ^1^, ^2^, ^3^ ^]^ The so‐called targeted covalent inhibitors (TCIs) and conceptually related modalities have proven to have advantages over their classic non‐covalent counterparts, such as a strong and persistent drug–target interaction, as well as high selectivity degree, if properly designed.^[^ ^4^, ^5^ ^]^ In recent years, pharmaceutical companies have brought several TCIs for different therapeutic applications to the market, for instance the irreversible covalent KRAS G12C inhibitor sotorasib (**1**),^[^ ^6^ ^]^ and also reversible‐covalent drugs like voxelotor (**2**)^[^ ^7^ ^]^ and nirmatrelvir (**3**)^[^ ^8^ ^]^ (**Figure** **1**A). Particularly in the protein kinase field, TCIs have proven to be a very fruitful strategy to obtain high‐quality chemical tools and successful drugs.^[^ ^1^, ^9^ ^]^ In most cases, these inhibitors are designed to bind to poorly conserved non‐catalytic amino acid residues, mainly cysteine (see, e.g., afatinib, **4**, and ibrutinib, **5**—Figure 1B). However, more highly conserved residues, such as the “catalytic” lysine (see, e.g., the reversible‐covalent inhibitors **6** and **7**—Figure 1C), can be addressed as well.^[^ ^10^ ^]^ To enable the covalent binding process, TCIs feature a reactive group, typically a weak electrophile, often called the covalent “warhead”, which is strategically attached to a reversibly binding, nonreactive molecular scaffold.^[^ ^11^, ^12^ ^]^ In addition to this, so‐called “electrophile first” approaches are becoming increasingly common.^[^ ^2^, ^13^ ^]^ For this kind of approach, electrophilic fragments are screened and the identified hits are elaborated using fragment‐based or traditional drug discovery techniques. Generally, irreversible covalent inhibitors bind via a two‐step mechanism, with a first equilibrium step (described by the equilibrium constant *K*_i_) followed by the chemical reaction forming a covalent bond (defined by the rate constant *k*_inact_). Therefore, covalent binding is time dependent and best described by the kinetic parameter *k*_inact_/*K*_I_, a second‐order rate constant comprising the overall efficiency of the covalent binding process.^[^ ^14^, ^15^ ^]^ Importantly, *k*_inact_ is not the same as intrinsic chemical reactivity and these parameters are not necessarily correlated.^[^ ^12^ ^]^

**Scheme 1:**
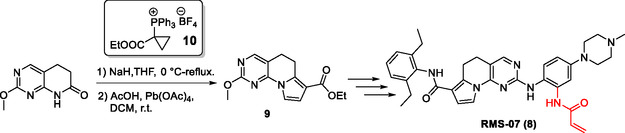
Key step in the synthesis of **RMS‐07 (8)** limited by the availability of phosphonium reagent **10** (from ref. [16]). Warhead group highlighted in red.

In 2022, our research group disclosed **RMS‐07** (**8**, **Scheme** **1**),^[^ ^16^ ^]^ a First‐in‐Class covalent inhibitor of the protein kinase monopolar spindle 1 (MPS1, also known as dual specificity protein kinase threonine tyrosine kinase (TTK)). This compound targets a poorly conserved cysteine (Cys604) at the kinase's middle hinge region (gatekeeper (GK)+2 position) via its acrylamide moiety. Notably, most of the other kinases featuring an equivalently positioned cysteine (i.e., FGFR4, MAPKAPK2 and 3, and S6K2) have already been covalently addressed with highly efficient covalent inhibitors, using different types of electrophilic warheads.^[^ ^17^, ^18^, ^19^, ^20^, ^21^ ^]^ In contrast, as shown by us and others,^[^ ^16^, ^22^ ^]^ achieving fast covalent binding (i.e., high *k*_inact_) is more difficult in MPS1. Presumably, this is due to a protein microenvironment promoting a lower nucleophilicity of the target cysteine or due to differences in the orientation of the hinge loop, which results in a nonideal alignment of the cysteine and the warhead within the pre‐reaction complex.

MPS1 plays a central role in the chromosome segregation process, being responsible for the maintenance of the proper function of the spindle assembly checkpoint, which is also known as the mitotic checkpoint.^[^ ^23^, ^24^ ^]^ Studies have strongly associated MPS1 overexpression with development of solid tumors, such as triple negative breast cancer,^[^ ^25^ ^]^ glioblastoma,^[^ ^26^ ^]^ and pancreatic cancer.^[^ ^27^ ^]^ Furthermore, the kinase has recently been identified as a novel prognostic marker for neuroblastoma.^[^ ^28^ ^]^ Inhibitor **8** shows nanomolar potency against MPS1 in vitro and target engagement in living cells, as well as a high kinome selectivity and promising results in a variety of different solid cancer cell lines. Nevertheless, its relatively slow inactivation kinetics (*k*_inact_ = 2.42 × 10^−4^ s^−1^) require optimization.^[^ ^16^ ^]^ To elucidate structure–activity relationships (SAR) and structure–kinetic relationships, it is necessary to synthesize a range of analogues of compound **8**. Therefore, a scalable route for the tricyclic scaffold **9** (Scheme 1) is required. While such a synthesis has been published^[^ ^29^, ^30^, ^31^ ^]^ and optimized to meet the setup in an academic medicinal chemistry lab,^[^ ^16^ ^]^ synthetic access to scaffold **9** was limited by the price and availability of the key reagent [1‐(ethoxycarbonyl)cyclopropyl] triphenylphosphonium tetrafluoroborate (**10**), which is used to assemble the third annulated ring via a Wittig‐type cyclization reaction. The impact of the price and availability of reagent **10** is particularly pronounced because of the poor atom economy of Wittig reactions, necessitating the use of more starting material and therefore further emphasizing the importance of a reliable access to this material. In the context of our synthesis route, this precious reagent can be regarded as the “gold”. While a previous report from the 1970s described the synthesis of the reagent,^[^ ^32^ ^]^ the published procedure contains no experimental details and could not be reproduced in our laboratories, thus impeding the scale‐up of the compound **8** scaffold synthesis.

**Figure 2:**
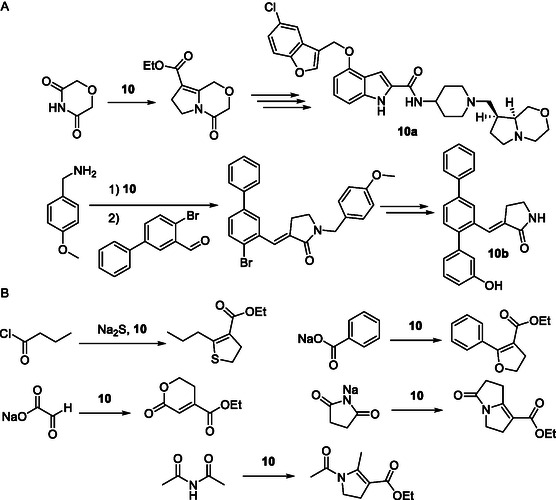
Examples of other applications (A and B) for the reagent **10**.

Herein, we successfully established and optimized a concise and reproducible protocol that facilitates decigram‐scale access to the key reagent [1‐(ethoxycarbonyl)cyclopropyl] triphenylphosphonium tetrafluoroborate (**10**), required for the synthesis of novel and more efficient covalent MPS1 inhibitors derived from compound **8**. Our protocol will also be of value to other medicinal chemistry programs, which rely on the availability of larger quantities of reagent **10** for building up bioactive ring systems. For instance, the bicyclic morpholine in the C‐C chemokine receptor type 2 and 5 (CCR2/CCR5) antagonist **10a** by Novartis^[^ ^33^ ^]^ and the substituted pyrrolidone in the anti‐fibrotic agent **10b** by Vectus Biosystems^[^ ^34^ ^]^ (**Figure** **2**A) illustrate applications of reagent **10**. Moreover, reagent **10** can be employed as a useful synthon for the synthesis of a variety of substituted ring systems with an application potential in drug discovery, including dihydrothiophenes,^[^ ^35^ ^]^ dihydrofurans,^[^ ^36^ ^]^ dihydropyrones,^[^ ^37^ ^]^ pyrrolizidinones,^[^ ^38^ ^]^ and dihydropyrroles^[^ ^39^ ^]^ (Figure 2B).

## 2 Results And Discussion

### 2.1 Chemistry

**Scheme 2:**
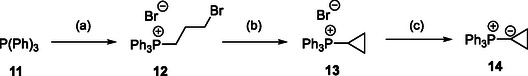
Synthetic route leading to the phosphonium bromide salt intermediate **13** and the following formation of the ylide **14** under basic conditions. Reagents and conditions: a) 1,3‐dibromopropane (1.05 eq), toluene (0.9 m), at 115 °C, for 16 h, yield: 62%; b) NaOH aq. (1 m), at 100 °C, for 20 h, quantitative yield. c) LDA (4 eq), THF, at −20 °C.

The synthesis of reagent **10** commenced with the preparation of the intermediate cyclopropyl phosphonium bromide **13** (**Scheme** **2**) according to an experimental procedure from González et al.^[^ ^40^ ^]^ In this synthesis, which could easily be conducted at multigram scale in excellent yield, 1,3‐dibromopropane was reacted with triphenylphosphine to generate the intermediate (3‐bromopropyl)triphenylphosphonium salt **12**, which was subsequently deprotonated to form the cyclopropane ring of **13** via an intramolecular S_N_2 reaction. The synthesis of [1‐(ethoxycarbonyl)cyclopropyl] triphenylphosphonium tetrafluoroborate **10** described by Fuchs^[^ ^32^ ^]^ relies on deprotonation of phosphonium salt **13** bearing a bromide as the counterion, and a subsequent ethoxycarbonylation reaction of the ensuing ylide (**14**) with ethyl chloroformate (ECF) as the electrophile, followed by a tetrafluoroborate ion exchange. However, initially we faced solubility issues with phosphonium bromide salt **13** in different organic solvents, leading to incomplete conversion and the formation of undefined products, even when large excess of different bases and ECF was used (Table S1, Supporting Information).

While little conversion was observed with commercial lithium diisopropylamide (LDA), sodium hydride, or butyllithium (the integrity of the bases was confirmed by use in other reactions), the suspension of **13** in tetrahydrofurane (THF) showed a slight color change toward yellow upon addition of 1 eq of freshly prepared LDA, with the color becoming more prominent with more equivalents of LDA being added. After addition of 4 eq of LDA, the suspension was of an intense brown color, suggesting the formation of the expected ylide intermediate **14** after deprotonation. However, at this point, there was still some undissolved starting material **13** present. The addition of ECF after 25 min leads to another color change, with the brown color fading over the course of 30 mins to result in a yellow reaction mixture with some undissolved colorless solid remaining. Analysis of the reaction mixture using thin layer chromatography–mass spectrometry (TLC–MS) showed formation of a compound with the expected mass of the product **15** (375 Da). However, at this point, there was still a large amount of unconverted precursor **13** present as shown by MS and nuclear magnetic resonance (NMR).

Importantly, we encountered significant variabilities during our optimizations that we could trace back to the quality of the individual reagents used. Thus, to ensure reproducibility, measures need to be taken to ensure a high purity of all reagents employed. This includes the distillation of diisopropylamide (DIPA) used for LDA preparation over NaOH and subsequent storage over molecular sieves (3 Å), the use of high‐quality *n*‐BuLi and determination of its concentration by titration as well as washing ECF with water followed by distillation, and a subsequent storage over molecular sieves (3 Å). Those precautions resulted in the clean (yet incomplete; see later) formation of ylide **14** after addition of only 1 equivalent of LDA and mitigated the generation of by‐products.

Despite the measures taken to ensure the quality of the reagents used, the undissolved starting material **13** remained a problem, resulting in purity issues and problems during the following ion exchange to replace halide counterion with BF_4_^−^. Sonication and meticulous grinding of the staring material **13** did not produce satisfactory solubilization. Even after using different BF_4_^−^ sources and several isolation/purification methods (**Table** **1**), including the recrystallization with diethyl ether/chloroform described in the original paper,^[^ ^32^ ^]^ we did not succeed in obtaining the desired final product **10** in acceptable quality.

**Table 1:** Attempts toward BF_4_^−^ ion exchange.

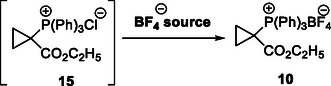		
Entry	BF_4_^−^ source	Isolation/purification method
1	NaBF_4_ direct addition of the solida)	Filtration
2	NaBF_4_ direct addition of the solid	Recrystallization with diethyl ether and chloroform or dichloromethane (DCM)
3	NaBF_4_ aqueous solutions (0.5–2.5 m)	Extraction with DCM followed by recrystallization with diethyl ether and chloroform
4	NaBF_4_ aqueous solutions (0.5–2.5 m)	Extraction with DCM followed by flash chromatography with DCM/MeOH
5	Meerwein's reagent (triethyloxonium tetrafluoroborate in DCM solution, 1.0 m)	Filtration

a) **15** was used as obtained from the previous step without isolation.

**Scheme 3:**
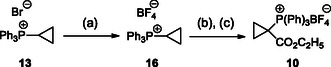
Optimized decigram synthetic route of the key reagent **10**. Reagents and conditions: a) NaBF_4_ aq (7.5 m), DCM (0.6 m), at room temperature, for overnight, yield: 82%; b) LDA (1.1 eq), THF (0.24 m), at −20 °C, for 20 min, without isolation; c) ethyl chloroformate (1.1 eq.) in THF 1:5, at −78 to −96 °C, for 55 min, yield: 86%, two steps.

The solubility issues could, however, be greatly improved by performing an ion exchange with NaBF_4_ in the first step, unlike previously described,^[^ ^32^ ^]^ to form tetrafluoroborate intermediate **16** (**Scheme** **3**). This BF_4_^−^ salt has significantly increased solubility in organic solvents in comparison to the Cl^−^ salt **15**. Still, performing the ion exchange as the first step alone was not sufficient to achieve full solubilization within 1 h after LDA addition. Finally, this problem could be resolved by fine grinding and thorough drying of the freshly prepared BF_4_^−^ intermediate **16** after isolation. This resulted in complete dissolution of the starting material and formation of a dark red solution 15 min after adding the LDA at −20 °C. Subsequent addition of purified ECF leads to an instant precipitation of **10** as a colorless solid (Scheme 3). This precipitate, however, had a peculiar nature as it tended to aggregate, taking up a state which clogs up glass‐fritted and paper filters, as well as hindering the stir bar from stirring properly. This aggregate could only be disintegrated by extensive sonication. Aggregation could be prevented by adding the ECF very slowly and ensuring throughout mixing of the suspension. The precipitate was very voluminous and stirring with standard magnetic stirrers proved to be challenging, limiting the synthesis to a scale of ≈3–4 g.

Even when taking great care to prevent the stir bar from getting stuck, the filtration of the precipitate itself also proved to be challenging. Upon addition of the reaction mixture to a glass‐fritted filter funnel for vacuum filtration, the mixture tended to block the filter and extended time needed for filtration caused the formation of a slimy residue on the filter. While the formation of this slimy material depended on several variables, the most central aspect was the flow rate that could be achieved in the filter. Generally, a smaller diameter and smaller pore size of the filter increased the likelihood of clogging of the filter. As a guideline, on the 4 g scale, a 125 mL filter with a porosity class 3 and membrane pump vacuum proved to be able to reproducibly filter off the precipitate without the filter getting blocked. However, an increased synthesis scale (>4 g) of **10** could not be reliably carried out anymore, when using a magnetic stirrer and the 125 mL filter.

To solve the problem of scaling up, we employed a setup with a mechanical stirrer in a 1L three‐necked flask. The mechanical stirrer had no problem keeping the thick suspension that formed upon ECF addition vigorously stirring and enabled a successful outcome of the reaction up to a scale of 32.5 g (Figure S10, Supporting Information). Although we did not test larger quantities, the experience gathered suggests that scaling up by at least another factor 2 would not be an issue with this setup. To further lower the solubility of the precipitating product **10** and in turn increase the yield isolated from the reaction, we reduced the amount of the THF solvent significantly and also cooled the reaction mixture down to −96 °C before adding it to the filter. The filter used for vacuum filtration was a 1L glass‐fritted filter funnel with a porosity class 3 (0.5 mbar; membrane pump vacuum). It effectively filtered off the precipitate without clogging up and achieved a very high flow rate.

Unfortunately, it was not possible to directly transfer all the precipitate into the filter funnel. Thus, after the filtration of the initial precipitate, the flask was rinsed with cold THF (−70 °C) to suspend the remaining precipitate in the flask for a second filtration.

At this point, the product still contained lithium halides and potentially LiOH which may form from excess LDA reacting with water from the air during the filtration. While these inorganic solids are poorly soluble in dichloromethane (DCM), the desired product, BF_4_^−^ salt **10**, has a high solubility in DCM, which enabled separation by dissolution and filtration through a plug of celite. We successfully reproduced this synthesis multiple times on varying scales, with the highest yield achieved being 92% on a 1 g scale. On the 32.5 g scale (Figure S10, Supporting Information, Scheme 3 and Scheme S1, Supporting Information), the yield was 86% and the product was obtained at an high‐performance liquid chromatography (HPLC) purity of >95% (Figure S8, Supporting Information). Notably, we have already used the obtained material in the subsequent cyclization reaction toward compound **9** (see Scheme 1),^[^ ^16^ ^]^ where it showed equivalent or better performance as the commercial material.

**Figure 3:**
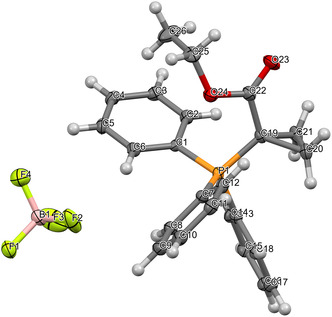
Structure of **10** determined by X‐ray crystallography. The crystal further confirms the desired structure and the presence of BF_4_^−^ as the counterion. Crystal data and structure refinement are shown in Table S2, Supporting Information. The structure was deposited in the CCDC database under the number 2,390,029.

To confirm the full retention of the BF_4_^−^ counterion throughout the synthetic route, quantitative ^19^F‐NMR studies were conducted. Initially, hexafluorobenzene was used as an internal standard. However, due to its slightly volatile nature, it proved challenging to perform an accurate quantification of the synthesized product, as well as of the commercial compound used for validation. To increase the accuracy, the less volatile 4’‐(trifluoromethyl)acetophenone was chosen as a new standard. Therefore, we quantified the amount of BF_4_^−^ counterion present before and after performing the ethoxycarbonylation reaction. We found full retention of the counterion with the ratio of BF_4_^−^ after the reaction still being >97% (Figure S7, Supporting Information). Moreover, to obtain a full structural characterization of the desired compound, we determined the X‐ray crystal structure of **10** (**Figure** **3**; see Experimental Section for crystallization conditions). The data undoubtedly confirmed the molecular structure and the presence of BF_4_^−^ as the counterion.

## 3 Conclusion

In this study, we established and optimized a reproductible decigram‐scale synthesis protocol for [1‐(ethoxycarbonyl)cyclopropyl] triphenylphosphonium tetrafluoroborate (**10**), a limiting key reagent and thus the “gold” in the synthesis of covalent MPS1 inhibitor **8** and its analogs. Different challenges were overcome, such as the lack of a detailed experimental procedure in the literature, variabilities caused by impurities in commercial‐grade reagents, and the low organic solubility of the intermediate phosphonium salt **13** used as the starting material in the pivotal deprotonation/ethoxycarbonylation reaction. Handling techniques were optimized to mitigate the formation of aggregates of the precipitated product in the reaction flask and during filtration. Also, the great importance of previously purifying the used reagents is emphasized. Ultimately, we were able to obtain the desired final product **10** (32.5 g scale) as a pure colorless solid (>95% by HPLC) in a high yield (86%) from the reaction mixture though simple filtration steps, with no need for further purification via recrystallization or chromatographic methods. We fully characterized the obtained product by ^1^H and ^13^C‐NMR, high‐resolution mass spectrometry (HRMS) as well as quantitative ^19^F‐NMR to undoubtedly confirm the retention of the BF_4_^−^ counterion throughout the deprotonation/ethoxycarbonylation step, which is further confirmed by its molecular structure elucidated by X‐ray crystallography. In summary, we outline a scalable approach for synthesizing this expensive reagent in a standard academic medicinal chemistry lab, thereby enabling the exploration of SAR around the First‐in‐Class covalent MPS1 inhibitor **8**.

## 4 Experimental Section

4.1

4.1.1

##### Chemistry: General

All starting materials and reagents were of commercial quality unless the method for purification is mentioned later. Specifically, the solution of n‐BuLi was sourced from Sigma Aldrich (230,707–100mL). TLC was carried out on Merck 60 F254 silica plates (Merck KGaA, Darmstadt, Germany) and spots were visualized under UV light (254 and 366 nm) or developed with an appropriate staining reagent.

NMR spectra were recorded on Bruker Avance 400 instruments (Bruker Corporation, Billerica, MA, USA). The samples were dissolved in deuterated solvents and chemical shifts were given in ppm in relation to tetramethylsilane. Spectra were calibrated using the residual proton or carbon peaks of the used solvent. In the case of ^19^F NMR, 4’‐(trifluoromethyl)acetophenone was used as the reference. MS was carried out with an Advion TLC–MS interface (Advion, Ithaca, NY, USA) with electron spray ionization (ESI) in positive and/or negative mode. Instrument settings were as follows: ESI voltage of 3.50 kV, capillary voltage of 187 V, source voltage of 44 V, capillary temperature of 250 °C, desolvation gas temperature of 250 °C, and gas flow of 5 L min^−1^ nitrogen. HRMS for the final compound was measured by the MS department, Institute of Organic Chemistry, Eberhard Karls University Tuebingen on a Bruker maXis 4 G ESI‐time of flight (TOF). The instrument was run in ESI + mode, settings were as follows: nebulizer gas of 1.2 bar, gas flow of 6.0 L min^−1^, source temperature of 200 °C, capillary voltage of +4500 V, end plate offset of −500 V, and m/z range from 80 to 1000 m/z.

Purity of the final compound was determined via NMR and HPLC using an Agilent 1100 Series LC system (Agilent Technologies, Santa Clara, CA, USA) with a Phenomenex Kinetex C8 100 A column (150 × 4.6 mm, 2.6 μm) (Phenomenex Inc. Torrance, CA, USA) and detection was performed with a UV DAD at 254 nm and 230 nm wavelength. Elution was carried out with the following gradients: 0.01 m KH_2_PO_4_, pH 2.32 (solvent A), MeOH (solvent B). 0 min: 40% B/60% A, 9 min: 95% B/5% A, 10 min: 95% B/5% A, 11 min: 40% B/60% A, 16 min: 40% B/60% A, and flow 0.5 mL min^−1^. The final compound showed a purity above 95% according to the peak areas at the two different wavelengths.

##### Synthesis Procedures: (3‐Bromopropyl)triphenylphosphonium Bromide (12)

Experimental procedure followed according to González et al.^[^ ^40^ ^]^ In a 500 mL two‐necked flask connected to a reflux condenser, PPh_3_ (50.0 g, 190.4 mmol, 1.0 eq) was dissolved in toluene (200 mL) and stirred vigorously under argon atmosphere. Subsequently, 1,3‐dibromopropane (20.4 mL, 40.4 g, 200 mmol, 1.05 eq) was slowly added to the solution and the mixture was kept stirring for 16 h at 115 °C. After cooling down to room temperature (RT), the colorless solid formed was filtered in vacuum and washed with toluene. The desired intermediate **12** was obtained upon drying as a colorless solid in 62% yield (54.2 g). ^1^H NMR (400 MHz, DMSO) *δ* 7.94–7.89 (m, 3H), 7.86–7.75 (m, 12H), 3.77–3.62 (m, 4H), and 2.13–2.01 (m, 2H) (Figure S1, Supporting Information). ^13^C NMR (101 MHz, DMSO) *δ* 135.0 (d, *J* = 2.8 Hz), 133.5 (d, *J* = 10.2 Hz), 130.2 (d, *J* = 12.5 Hz), 118.1 (d, *J* = 86.1 Hz), 33.2 (d, *J* = 22.4 Hz), 25.6, 20.0 (d, *J* = 54.7 Hz) (Figure S2, Supporting Information). MS (ESI) m/z: 383.3 [M]^+^. HPLC *t*_ret_: 7.87 min.

##### Synthesis Procedures: Cyclopropyltriphenylphosphonium Bromide (13)

Experimental procedure followed according to González et al.^[^ ^40^ ^]^ In a 250 mL round‐bottom flask connected to a reflux condenser, the intermediate **12** (54.2 g, 116,8 mmol, 1.0 eq) was suspended in an aqueous NaOH solution (1 m, 116.80 mL, 1.0 eq). The mixture was kept stirring for 20 h at 100 °C. After cooling down to RT, CHCl_3_ (100 mL) was added and the layers were separated after extraction. The aqueous phase was extracted three more times with CHCl_3_ (150 mL each). The combined organic phase was dried over Na_2_SO_4_, filtered, and concentrated under reduced pressure. To the initially formed oil, Et_2_O was slowly added until a cloudy solution formed. After concentration of the material under reduced pressure, the desired intermediate **13** was obtained as a colorless solid in a quantitative yield (44.7 g). ^1^H NMR (400 MHz, CDCl_3_) *δ* 7.83–7.76 (m, 9H), 7.70–7.65 (m, 6H), 3.60–3.51 (m, 1H), 1.84–1.79 (m, 2H), 0.60–0.54 (m, 2H) (Figure S3, Supporting Information). ^13^C NMR (101 MHz, CDCl_3_) *δ* 135.2 (d, *J* = 2.7 Hz), 133.8 (d, *J* = 9.8 Hz), 130.4 (d, *J* = 12.6 Hz), 118.5 (d, *J* = 89.5 Hz), 5.1 (d, *J* = 4.9 Hz), 0.7 (d, *J* = 86.6 Hz) (Figure S4, Supporting Information). MS (ESI) m/z: 303.3 [M]^+^. HPLC *t*_ret_: 7.08 min.

##### Synthesis Procedures: Cyclopropyltriphenylphosphonium Tetrafluoroborate (16)

A flask was charged with intermediate **13** (9.00 g, 1 eq, 23.5 mmol) which was dissolved in DCM (40 mL). Then a solution of NaBF_4_ (20.6 g, 8 eq, 188 mmol) in H_2_O (25 mL) was added. The mixture was stirred at RT overnight. The phases were separated, and the aqueous phase was extracted three times with 30 mL of DCM. The organic phases were combined, dried over anhydrous Na_2_SO_4_, filtered, and concentrated under reduced pressure to afford 7.50 g of the product as a colorless solid (82%).^[^ ^41^ ^]^

##### Synthesis Procedures: Purification of DIPA

DIPA was refluxed over NaOH for 30 min and then distilled at 85 °C under argon atmosphere. The material was then stored in a septum‐sealed bottle over 3 Å molecular sieves.

##### Synthesis Procedures: Purification of ECF

The ECF was washed several times with water and then distilled at 95 °C using a fractionating column under argon atmosphere. The material was stored in a sealed bottle over 3 Å molecular sieves.

##### Synthesis Procedures: Preparation of LDA Solution

An oven‐dried 100 mL flask equipped with a stir bar was charged with dry THF (62 mL). Previously distilled DIPA (6 mL, 1 eq, 42.5 mmol) was added under positive argon pressure. The flask was cooled down to −78 °C. Then, n‐butyllithium (17 mL, 1 eq, 42.5 mmol) was added. The solution was stirred at −78 °C for 15 min to yield 85 mL of a 0.5 m solution of LDA in THF.

##### Synthesis Procedures: [1‐(Ethoxycarbonyl)cyclopropyl]triphenylphosphonium Tetrafluoroborate (10)

An oven‐dried 1L three‐necked flask equipped with a mechanical stirrer was charged with intermediate **16** (32.46 g, 1 eq, 84.7 mmol) under positive argon pressure. It was important to ensure that the material was dry and of a small enough particle size to obtain adequate solubility. This was achieved by using a mortar to grind the solid exhaustively and subsequent drying in vacuo. Dry THF (360 mL) was added to the solid via canulation using positive argon pressure. The suspension was cooled down to −20 ° C after complete solvent addition. Then, the previously prepared LDA solution (84.6 mL, 1.1 eq, 42.3 mmol) was added dropwise at −20 °C via canulation at a rate that avoided a significant rise of the temperature. Upon formation of a dark red solution after 20 min, the reaction mixture was cooled down to −78 °C. Previously distilled and dried ECF (8.87 mL, 1.1 eq, 93.2 mmol) diluted 1:5 with dry THF was added slowly while carefully controlling the temperature leading to precipitation of the product and a color change from dark red to light yellow. Care must be taken that the stirrer keeps stirring the highly viscous reaction mixture vigorously to furnish a homogeneous suspension. After an additional 55 min, the suspension was cooled down to −96 °C using an acetone/liquid nitrogen bath and vacuum‐filtered with a 1L large glass‐fritted filter with porosity class 3 as quickly as possible. Solid remaining in the flask was recovered by rinsing with −70 °C cold THF and filtration. Then, redissolution in DCM and filtration through a plug of celite to remove insoluble impurities followed by solvent evaporation gave the pure product as colorless solid. The compound was dried overnight on a high vacuum pump to afford 33.5 g (86% yield) of the desired key reagent **10**. ^1^H NMR (400 MHz, CDCl_3_) *δ* 7.85–7.78 (m, 3H), 7.77–7.70 (m, 12H), 4.03 (q, *J* = 7.1 Hz, 2 H), 2.22–2.16 (m, 2H), 1.40–1.37 (m, 2H), 0.84 (t, *J* = 7.1 Hz, 3H) (Figure S5, Supporting Information). ^13^C NMR (101 MHz, CDCl_3_) *δ* 167.9 (d, *J* = 7.1 Hz), 135.5 (d, *J* = 3.1 Hz), 134.1 (d, *J* = 9.7 Hz), 130.6 (d, *J* = 13.0 Hz), 117.7 (d, *J* = 91.0 Hz), 63.5, 17.0 (d, *J* = 90.9 Hz), 16.2 (d, *J* = 1.6 Hz), 13.5. (Figure S6, Supporting Information). ^19^F NMR (376 MHz, CDCl_3_) *δ* −153.24 (s), −153.30 (s) (Figure S7, Supporting Information). Interestingly, the BF_4_^−^ counterion in ^19^F‐NMR gave a twin peak (see Supporting Information) because of the high natural abundance of both the ^10^BF_4_^−^ and the ^11^BF_4_^−^ isotopes.^[^ ^42^ ^]^ HRMS ESI‐TOF: m/z calculated for C_24_H_24_O_2_P [M]^+^: 375.15138. Found: 375.15122 (Figure S9, Supporting Information). HPLC *t*_ret_: 7.07 min (Figure S8, Supporting Information).

##### X‐Ray Crystallography

The crystals were obtained by two different methods: 1) vapor diffusing of diethyl ether into a 5 mg mL^−1^ solution of compound **10** in methanol (298 K, 1 atm) over 1 week; 2) overlaying a solution of 5 mg mL^−1^ of compound 10 with pentane (298 K, 1 atm). The intermixing of the solvents and the crystallization occurred within 4 weeks. Crystal data for C_24_H_24_BF_4_O_2_P (*M* = 462.22 g mol^−1^): monoclinic space group: P 212 121; *a* = 10.7427(3) Å, *b* = 13.3081(3) Å, *c* = 15.5381(4) Å; *α*/*β*/*γ* = 90°; *V* = 2221.40(10) Å^3^; *Z* = 4; *T* = 120 K; *μ* = 0.176 mm^−1^; *ρ*_calc_ = 1.382 Mg m^−3^; 10,210 reflections measured, 5277 unique [*R*_int_ = 0.0251], which were used in all calculations.

## Conflict of Interest

The authors declare no conflict of interest.

## Supporting information

Supplementary Material
