# Supplementary material for: Go for Gold: Development of a Scalable Synthesis of [1‐(Ethoxycarbonyl)cyclopropyl] Triphenylphosphonium Tetrafluoroborate, a Key Reagent to Explore Covalent Monopolar Spindle 1 Inhibitors
Source: ChemistryOpen. 2025 Apr 16;14(9):e202500106. doi: 10.1002/open.202500106 (PMC12409829; doi:10.1002/open.202500106)
Supplement: Supplementary file 1 — Supplementary Material [file OPEN-14-e202500106-s001.pdf]

Supporting Information

Go for Gold: Development of a Scalable Synthesis of [1-(Ethoxycarbonyl)cyclopropyl] triphenylphosphonium tetrafluoroborate, a Key Reagent to Explore Covalent MPS1 Inhibitors

Leon Rebhan[1,2], Rebekka Fürst[1,3,4], Dieter Schollmeyer[5], Ricardo A. M. Serafim[1,4,6]*, Matthias Gehringer[1,3,4]*

[1] Department of Pharmaceutical/Medicinal Chemistry, Institute of Pharmaceutical Sciences, Eberhard Karls University Tübingen, Auf der Morgenstelle 8, 72076 Tübingen, Germany

[2] Department of Chemistry, Biochemistry and Pharmacy, University of Bern, Bern, Switzerland

[3] Department for Medicinal Chemistry, Institute for Biomedical Engineering, Faculty of Medicine, Eberhard Karls University Tübingen, Auf der Morgenstelle 8, 72076 Tübingen, Germany

[4] Cluster of Excellence iFIT (EXC 2180) 'Image-Guided & Functionally Instructed Tumor Therapies', University of Tübingen, 72076 Tübingen, Germany

[5] Department Chemie, Zentrale Analytik, Johannes Gutenberg-Universität Mainz, Duesbergweg 10-14, 55099 Mainz, Germany

[6] Department of Organic and Pharmaceutical Chemistry, School of Engineering, Institut Químic de Sarrià (IQS), Universitat Ramon Llull (URL), Vía Augusta 390, 08017 Barcelona, Spain

*Shared correspondence:

ricardo.serafim@iqs.url.edu
matthias.gehringer@uni-tuebingen.de

Supporting Information

Contents

Supplementary Scheme S1 3
Supplementary Table 1 3
Supplementary Figure S1 4
Supplementary Figure S2 4
Supplementary Figure S3 5
Supplementary Figure S4 5
Supplementary Figure S5 6
Supplementary Figure S6 6
Supplementary Figure S7 7
Supplementary Figure S7A........ 7
Supplementary Figure S8 8
Supplementary Figure S9 9
Supplementary Figure S10 10
Supplementary Table 2 11

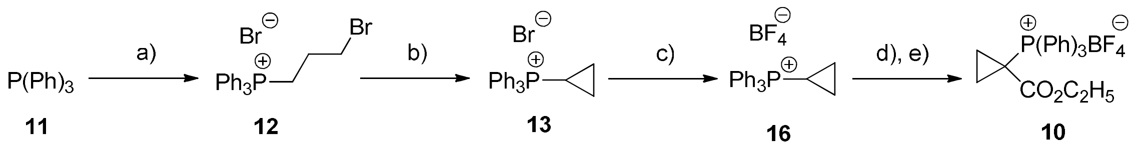

Supplementary Scheme S1 – Synthetic route leading to *(1-(ethoxycarbonyl)cyclopropyl) triphenylphosphonium tetrafluoroborate* (**10**). Reagents and conditions: a) 1,3-dibromopropane (1.05 eq), Toluene (0.9 M), 115 °C, 16 h, 62%; b) NaOH aq. (1 M), 100 °C, 20 h, quant. yield; c) $NaBF_4$ aq (7.5 M), DCM (0.6 M), r.t., overnight, 82%; d) LDA (1.1 eq), THF (0.24 M), -20 °C, 20 min, without isolation; e) Ethyl chloroformate (1.1 eq.) in THF 1:5, -78 °C to -96 °C, 55 min, 86% two-steps.

Supplementary Table 1 – Optimization of the ylide formation.

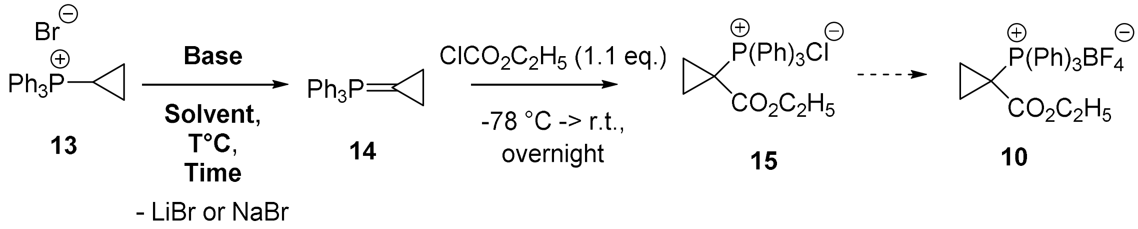

Entry	Base (equivalents)	Solvent (M)	Temperature (°C)	Time	Result (by HPLC and TLC-MS)
1	*LDA (1.05)	THF (0.3)	-20 → r.t.	3 h	No conversion
2	*LDA (1.05)	THF (0.005)	-20 → r.t.	3 h	No conversion
3	*LDA (1.05)	THF (0.005)	-20 → r.t.	on	No conversion
4	*NaH (1.0)	THF (0.3)	60	on	No conversion
5	*NaH (1.0)	THF (0.1)	60	on	No conversion
6	*NaH (1.0)	DMF (0.1)	60	on	No conversion
7	*n-BuLi (1.3)	THF (0.005)	-78 → r.t.	on	No conversion
8	#LDA (1.05)	THF (0.3)	-20	2 h	Partial conversion + undefined products

M = Molarity (reagent concentration in mol/L), r.t. = room temperature, on = overnight, *commercial source, #freshly prepared.

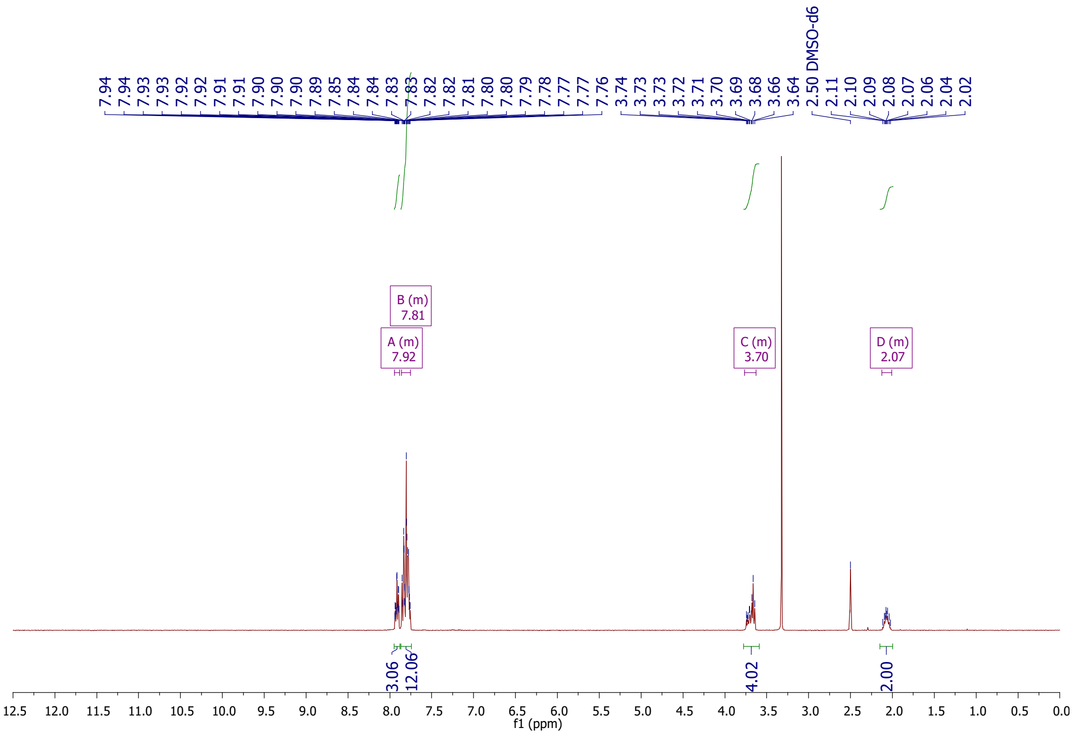

Supplementary Figure S1 – ^{1}H NMR (400 MHz, DMSO-d_6) of intermediate **12**.

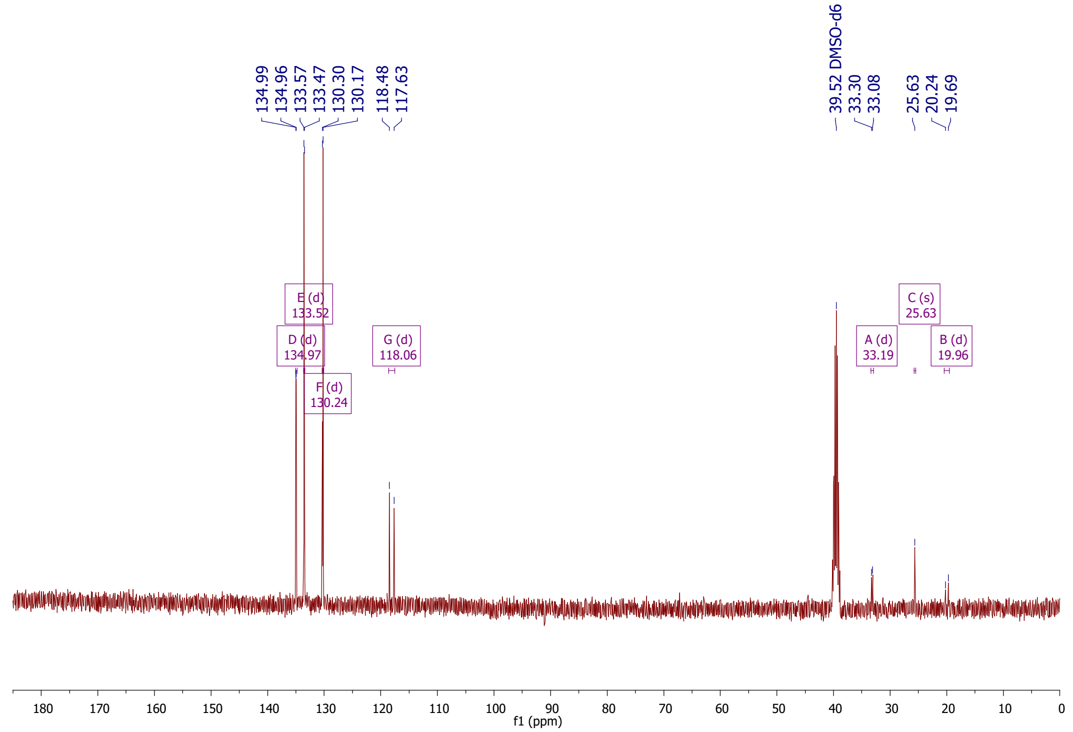

Supplementary Figure S2 – ^{13}C NMR (101 MHz, DMSO-d_6) of intermediate **12**.

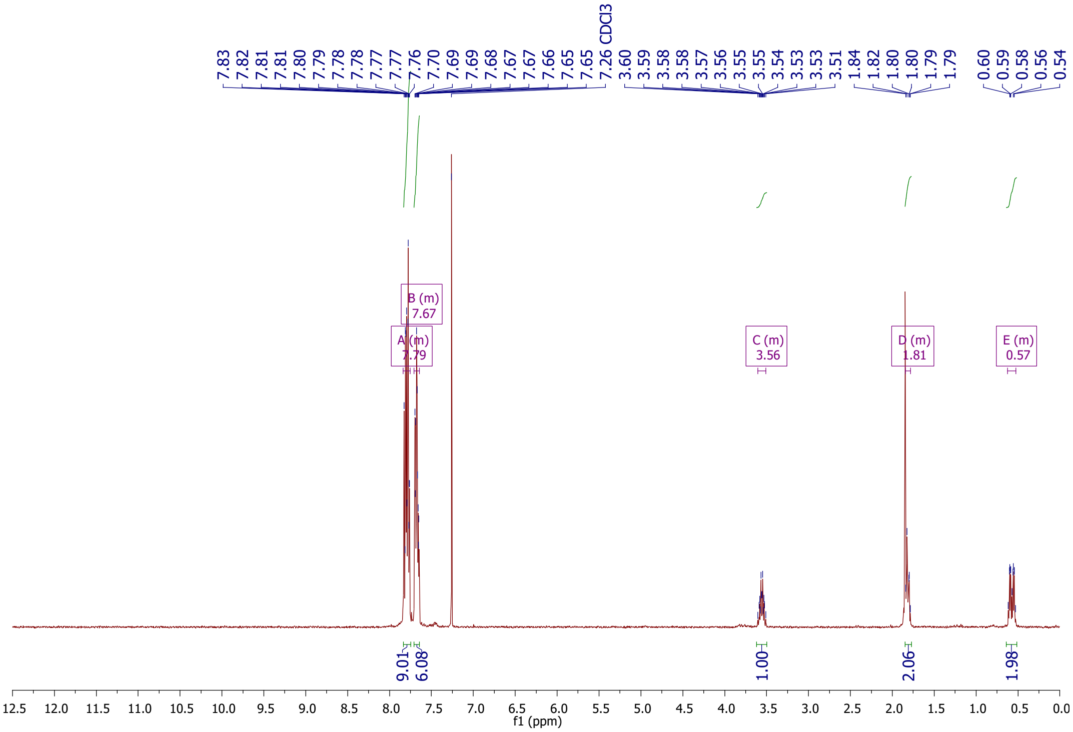

Supplementary Figure S3 – ^{1}H NMR (400 MHz, $CDCl_3$) of intermediate **13**.

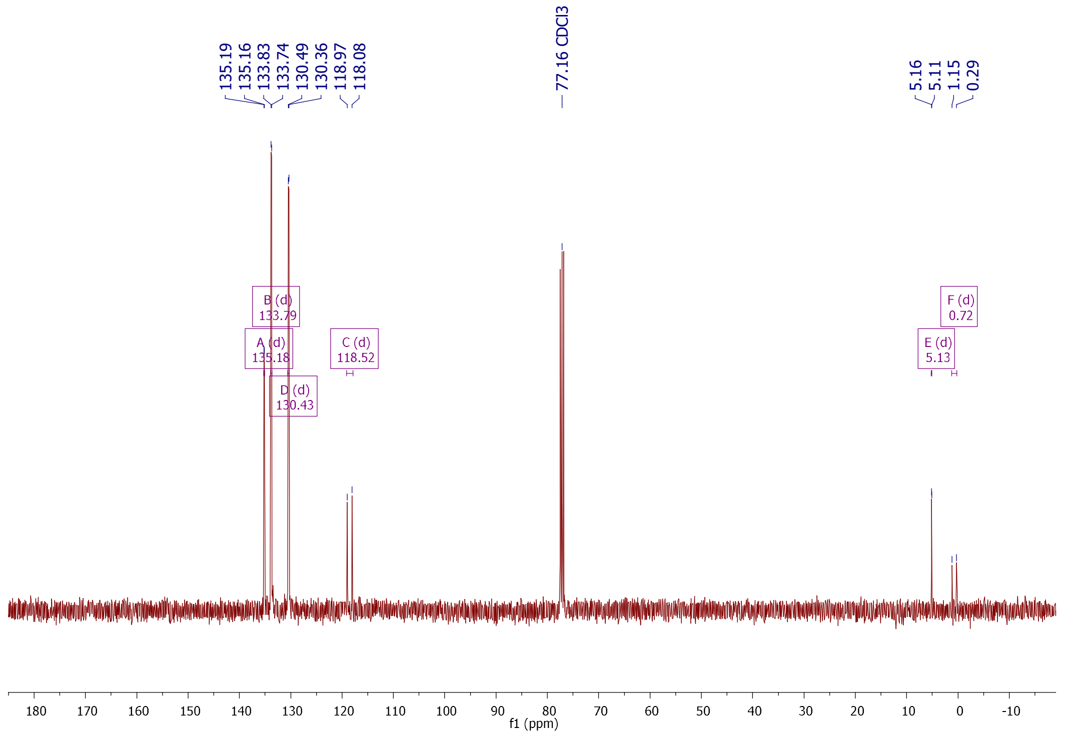

Supplementary Figure S4 – ^{13}C NMR (101 MHz, $CDCl_3$) of intermediate **13**.

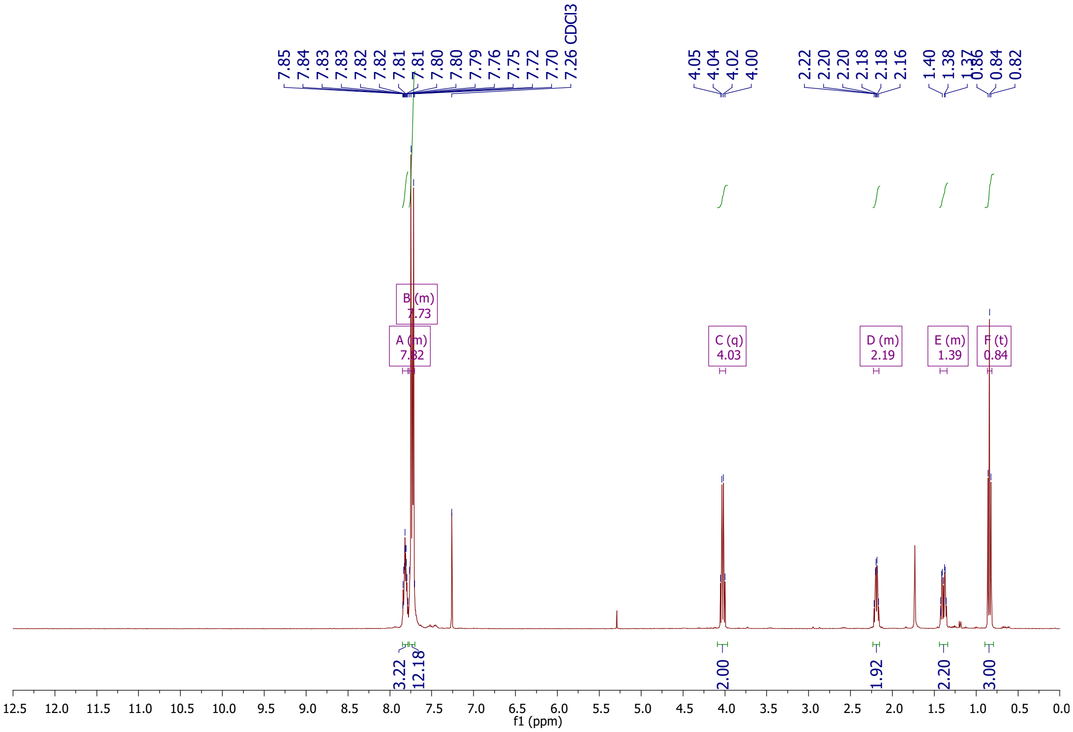

Supplementary Figure S5 – ^{1}H NMR (400 MHz, $CDCl_3$) of key reagent **10**.

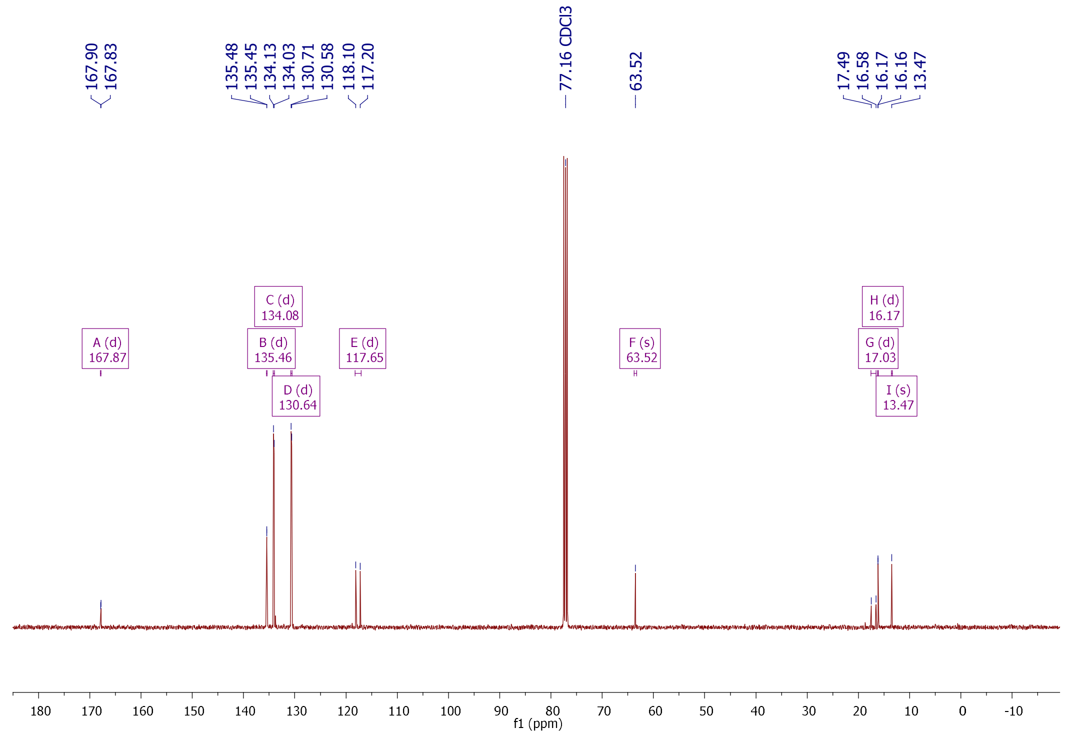

Supplementary Figure S6 – ^{13}C NMR (101 MHz, $CDCl_3$) of key reagent **10**.

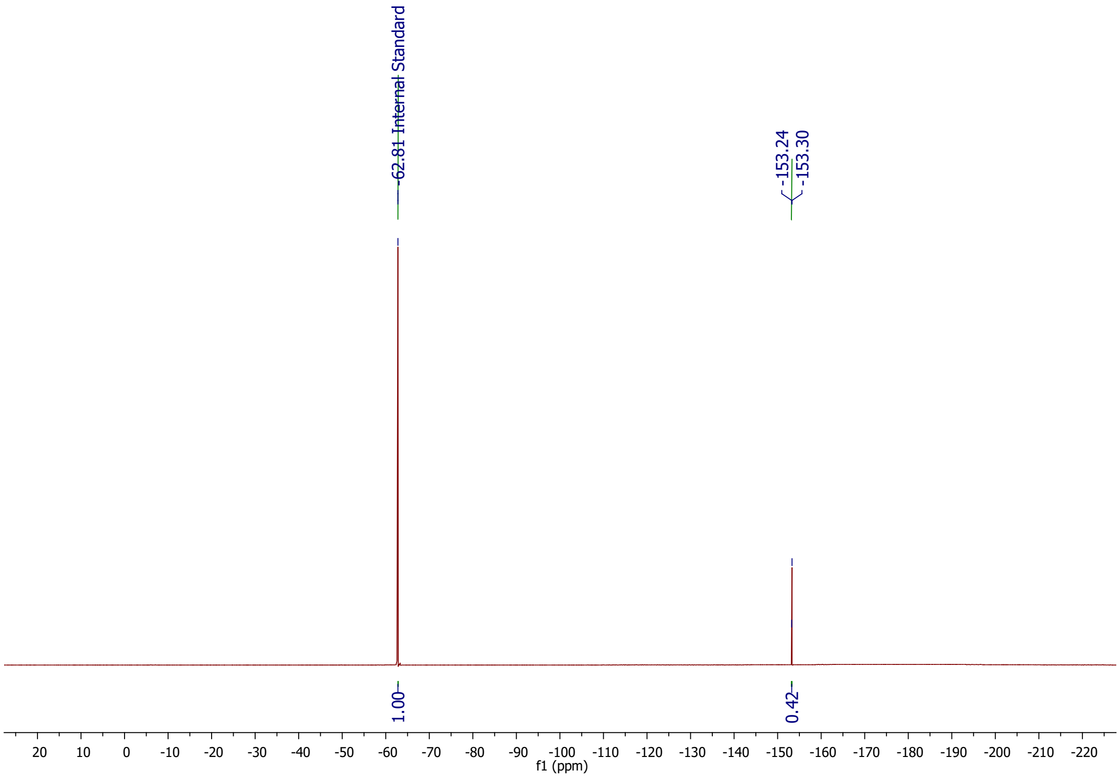

Supplementary Figure S7 – ^{19}F NMR (376 MHz, $CDCl_3$) of key reagent **10** with 4'-(trifluoromethyl)acetophenone as internal standard. Twin peak for **10** from ^{10}B and ^{11}B isotope.

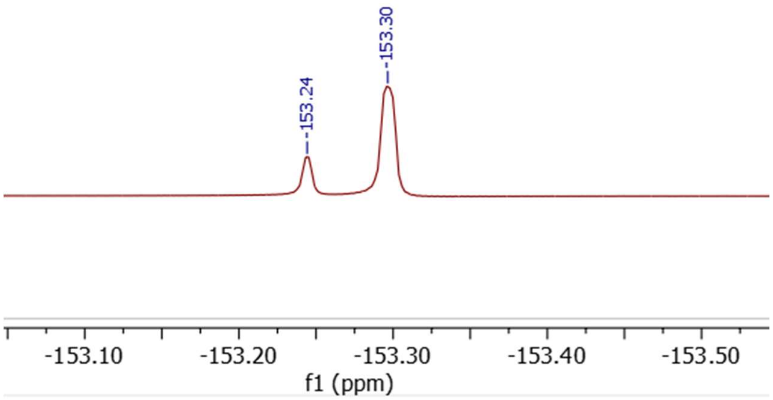

Supplementary Figure S7A

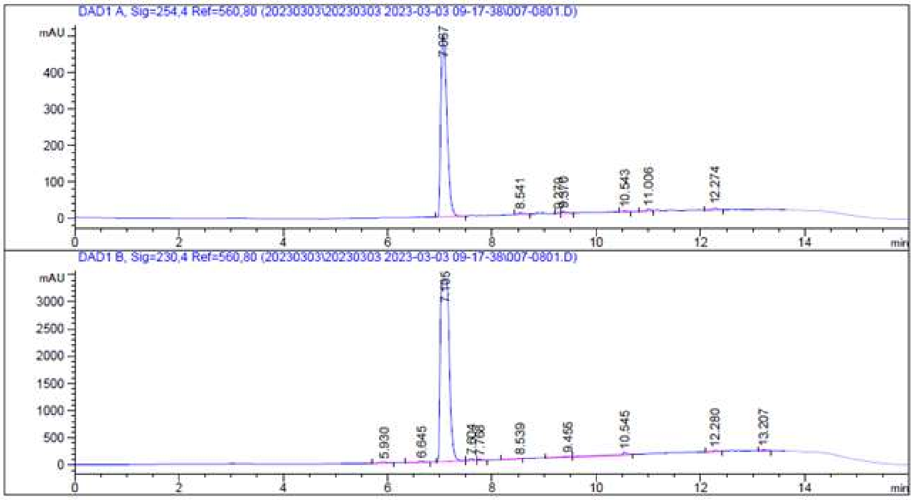

Area Percent Report

Sorted By : Signal
Multiplier: : 1.0000
Dilution: : 1.0000
Use Multiplier & Dilution Factor with ISTDs

Signal 1: DAD1 A, Sig=254,4 Ref=560,80

Peak #	RetTime [min]	Type	Width [min]	Area [mAU*s]	Height [mAU]	Area %
1	7.067	BB	0.1230	3980.58691	499.09988	96.0707
2	8.541	BB	0.0987	33.89960	4.67192	0.8182
3	9.279	BV	0.0596	12.87931	3.29254	0.3108
4	9.370	VB	0.0996	31.48388	4.29008	0.7599
5	10.543	BB	0.0731	11.13794	2.35717	0.2688
6	11.006	BV	0.0782	32.10312	6.02472	0.7748
7	12.274	BB	0.0996	41.30136	6.21079	0.9968

Totals : 4143.39214 525.94711

Signal 2: DAD1 B, Sig=230,4 Ref=560,80

Peak #	RetTime [min]	Type	Width [min]	Area [mAU*s]	Height [mAU]	Area %
1	5.930	BB	0.1418	153.53398	16.93245	0.3778
2	6.645	BB	0.1060	165.61732	23.57379	0.4075
3	7.105	BB	0.1640	3.88782e4	3335.48218	95.6650
4	7.604	BV	0.0875	206.33669	35.65945	0.5077
5	7.768	VB	0.0790	98.90885	18.31102	0.2434
6	8.539	BV	0.1254	107.69374	12.89752	0.2650
7	9.455	VV	0.1981	184.88324	12.11141	0.4549
8	10.545	VV	0.1690	614.52161	46.85374	1.5121
9	12.280	BB	0.1000	123.94489	19.02718	0.3050
10	13.207	MM	0.1219	106.30518	14.53723	0.2616

Totals : 4.06400e4 3535.38596

*** End of Report ***

Supplementary Figure S8 – HPLC traces of key reagent **10**.

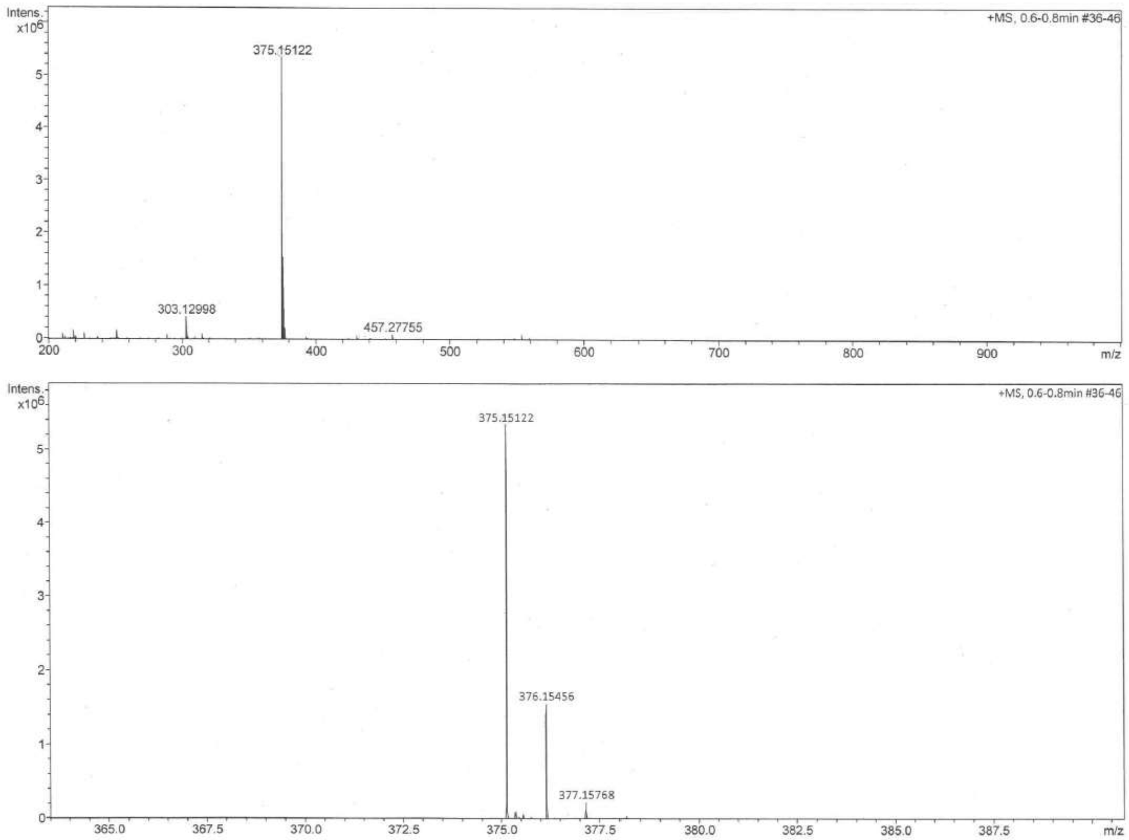

Supplementary Figure S9 – HRMS of key reagent **10**.

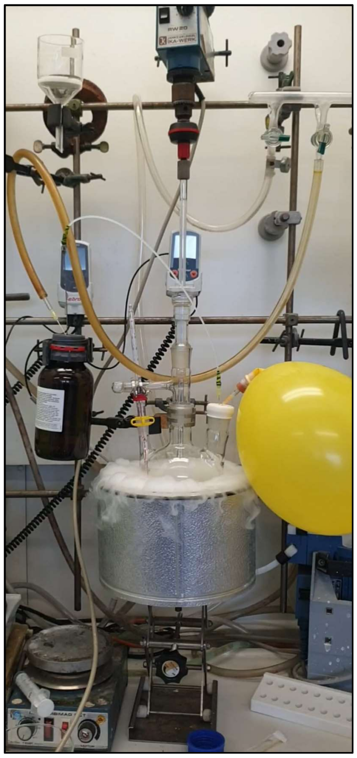

Supplementary Figure S10 – Image of the set-up of the 32.5 g scale synthesis of key reagent **10** in our laboratory.

Supplementary Table 2 – Crystal data and structure refinement for key reagent **10**.

CCDC Number	2390029	
Empirical formula	$C_{24}H_{24}BF_4O_2P$	
moiety formula	$C_{24}H_{24}O_2P$, BF_4	
Formula weight	462.21	
Temperature	120(2) K	
Wavelength, radiation type	0.71073Å, MoKα	
Diffractometer	STOE IPDS 2T	
Crystal system	Orthorhombic	
Space group name, number	P $2_12_12_1$, (19)	
Unit cell dimensions	a = 10.7427(3) Å	α = 90°
	b = 13.3081(3) Å	β = 90°
	c = 15.5381(4) Å	γ = 90°
Volume	2221.40(10) $Å^3$	
Number of reflections	16442	
and range used for lattice parameters	2.44° <=θ<= 28.39°	
Z	4	
Density (calculated)	1.382 Mg/m^3	
Absorption coefficient	0.176 mm^{-1}	
Absorption correction	Integration	
Max. and min. transmission	0.9756 and 0.9324	
F(000)	960	
Crystal size, colour and form colourless Block	0.210 x 0.400 x 0.470 mm^3,	
Theta range for data collection	2.437 to 27.886°.	
Index ranges 17<=l<=20	-12<=h<=14, -15<=k<=17, -	
Number of reflections:		
collected	10210	
independent	5277 [R(int) = 0.0251]	
observed [I>2sigma(I)]	5086	
Completeness to theta = 25.2°	99.9 %	

Refinement method	Full-matrix least-squares on F^2
Data / restraints / parameters	5277 / 0 / 290
Goodness-of-fit on F^2	1.070
Final R indices [I>2sigma(I)]	R1 = 0.0347, wR2 = 0.0892
R indices (all data)	R1 = 0.0366, wR2 = 0.0907
Absolute structure parameter	-0.01(5)
Largest diff. peak and hole	0.528 and -0.312 eÅ^{-3}
